# Effects of Dietary Rapeseed Meal on Growth Performance, Intestinal Structure, Gut Microbiota, and Related Gene Expression of Juvenile Largemouth Bass (*Micropterus salmoides*)

**DOI:** 10.3390/microorganisms13112535

**Published:** 2025-11-05

**Authors:** Ximing Hou, Haiqing Wu, Rongyan Yue, Xinghua Zhou, Yongjun Chen, Li Luo, Shimei Lin, Hailong Ge, Yuanfa He

**Affiliations:** 1 College of Fisheries, Southwest University, Chongqing 400715, China; 16638931670@163.com (X.H.); whq1543@163.com (H.W.); y18839407215@163.com (R.Y.); zxh6000@163.com (X.Z.); chenyjcq@swu.edu.cn (Y.C.); luoli1972@163.com (L.L.); linsm198@163.com (S.L.); 2 Key Laboratory of Freshwater Fish Reproduction and Development, Southwest University, Chongqing 400715, China; 3 Integrative Science Center of Germplasm Creation in Western China (Chongqing) Science City, College of Fisheries, Southwest University, Chongqing 400039, China

**Keywords:** fish meal replacement, intestinal permeability, histology, signaling pathway, transcriptome

## Abstract

This study aimed to explore the effects of dietary rapeseed meal replacing fish meal on growth performance, intestinal structure, gut microbiota, and related gene expression of juvenile largemouth bass (*Micropterus salmoides*). Five isonitrogenous and isolipidic diets were designed, in which rapeseed meal replaced 0% (FM, control), 5% (RM5), 10% (RM10), 15% (RM15), and 25% (RM25) of fish meal. Then, largemouth bass (11.00 ± 0.20 g) were randomly and equally allocated to 15 experimental tanks (25 fish per tank) for an 8-week feeding trial. The results showed that growth performance declined as replacement levels increased to 25%. However, the RM5 group had the highest body crude protein, distal intestinal muscle layer thickness (MLT), and plica height (PH) and width (PW), which were significantly higher than those of the FM group. In addition, compared to the FM group, the RM15 and/or RM25 groups had higher levels of D-lactic acid, diamine oxidase, and lipopolysaccharide. Furthermore, the RM25 group exhibited higher abundances of *Lactococcus* and *Weissella* but lower levels of *Aeromonas* and *Staphylococcus* compared to the FM group. Intestinal transcriptome analysis revealed that the PI3K-Akt and NF-κB signaling pathways were significantly up-regulated when comparing the RM25 and FM groups. The results demonstrate that the replacement of 5% fish meal with rapeseed meal did not have a negative impact on the physiological status of largemouth bass. However, a replacement level of 25% reduced growth performance and damaged intestinal structure, potentially by altering the abundance of intestinal microbiota and up-regulating the PI3K-Akt and NF-κB signaling pathways.

## 1. Introduction

The current global fish meal production cannot well meet the growing aquaculture industry, and the price of fish meal is expensive [1]. Consequently, exploring other protein sources that can replace fish meal has become an important task that urgently needs to be addressed at present. Researchers have evaluated the substitution of fish meal with various plant protein sources in aquatic feed, such as soybean [2,3], cottonseed [4,5], rapeseed [6,7], and lupine [8,9]. When numerous plant-based protein sources are used to substitute fish meal, they may cause stress reactions, intestinal inflammation, and exert pressure on the immune system [10,11]. This may be caused by the antinutritional factors in plant proteins [12]. Therefore, it is generally recognized that it is necessary to add a combined protein source to aquatic feed, that is, to mix animal protein and plant protein in a certain proportion, which can mitigate the damage caused by a large amount of plant protein [13]. Until now, being low in price, easily accessible, nutritionally balanced, and having a low content of antinutritional factors remain our requirements for the selection of plant proteins [14].

Rapeseed has been cultivated globally for millennia, primarily in Australia, Canada, China, and temperate regions of Europe, ranking as the third largest oilseed crop worldwide after oil palm and soybean [15]. Rapeseed meal, a by-product of rapeseed protein processing, can have a protein content ranging from 30% to 60% following processing [14]. Compared with fish meal, rapeseed meal has a higher yield, lower cost, and greater accessibility, and has been widely used in animal feed [16]. In addition, aquaculture has experienced rapid growth in recent years and has become an important branch of agriculture and a food production sector [17]. The rapeseed meal has a relatively balanced amino acid content, especially methionine and cysteine (1.60–1.61%), and it also contains high levels of vitamins, minerals, and other trace elements [18,19]. So far, rapeseed meal has been successfully applied to the feed for carnivorous and omnivorous fish in the aquaculture industry. It has been proven that it is feasible to replace a certain proportion of fish meal with rapeseed meal in red sea bream (*Pagrus major*) [20], yellow catfish (*Tachysurus fulvidraco*) [21], rainbow trout (*Oncorhynchus mykiss*) [22], and hybrid sturgeon (*Acipenser baerii* ♀ × *Acipenser schrenckii* ♂) [23]. It is worth noting that feeding yellow catfish with a diet containing 10% rapeseed meal had no negative effects on their specific growth rate (SGR) and feed conversion ratio (FCR). In addition, feeding red sea bream with fermented rapeseed meal effectively improved feed utilization, protein efficiency ratio (PER), and weight gain rate (WGR).

The gut microbiota not only regulates host health and prevents inflammatory bowel disease (IBD) [24], but also plays a role in the digestion and absorption of nutrients [25]. Different dietary patterns lead to variations in the composition of the host gut microbiota, and there is an inseparable relationship between diet, gut microbiota, and intestinal inflammation [26]. In addition, High-throughput sequencing can identify and classify complex microbial communities, thereby helping us better understand the relationship between the gut microbiota and intestinal diseases [27]. RNA sequencing, as a powerful tool for transcriptome analysis, enables us to efficiently quantify differentially expressed genes, discover novel transcripts, and identify alternatively spliced genes through high-throughput sequencing [28]. Multi-omics analysis has been applied in some fish species, and it can better help us understand how the gut microbiota and gene expression affect the phenotypes of fish [29,30].

Largemouth bass (*Micropterus salmoides*), belonging to the species *Micropterus salmoides* of the genus *Micropterus*, family Centrarchidae, order Perciformes, has become a major species in freshwater aquaculture in China due to the characteristics of fast growth, delicious meat, and high market demand [31]. The annual production of largemouth bass in China reached 888,030 tons in 2023 [32], and the market value of bass is approximately $3.52 per 500 g. In addition, the fish species is a carnivorous fish, and its dietary protein requirement is generally higher than that of other fish species [33], with 48–51% being the suitable range [34,35,36]. As an important source of protein, fish meal is widely used in the feed for largemouth bass. Meanwhile, rapeseed meal, a high-quality plant protein source, has been rarely reported in studies regarding its application in largemouth bass feed. Thus, this study aims to explore the impacts of feeding largemouth bass with feeds in which fish meal is replaced by rapeseed meal at different ratios. Through this exploration, it seeks to cut down on aquaculture costs, boost economic returns, and drive the sustainable development of the aquaculture industry.

## 2. Materials and Methods

### 2.1. Experimental Diets

Five isonitrogenous and isolipidic diets were designed, in which rapeseed meal replaced 0%, 5%, 10%, 15%, and 25% of fish meal, designated as FM (control), RM5, RM10, RM15, and RM25, respectively. The detailed ingredient compositions are provided in Table 1. Lysine and methionine were supplemented to meet the requirements of juvenile largemouth bass according to previous studies [37]. All ingredients were ground and sieved through a 60-mesh sieve, then accurately weighed according to the formula. Trace components were mixed uniformly using a gradual dilution method, followed by blending with other ingredients. The mixture was processed into pellet feeds with particle sizes of 2.0 mm and 2.5 mm using a twin-screw extruder (SG-YPYS-76, Xiamen Xinyuanfa Machinery Equipment Factory, Xiamen, China). The pellets were air dried at room temperature to a moisture content of approximately 8%, packed in sealed bags, and stored at −20 °C in a refrigerator for later use.

### 2.2. Experimental Procedures

All the juvenile largemouth bass were purchased from Chongqing Three Gorges Ecological Fishery Co., Ltd. (Chongqing, China) and were acclimated for two weeks using commercial feed (Foshan Jieda Feed Co., Ltd., Foshan, China). At the start of the experiment, fish were fasted for 24 h, anesthetized with 0.1 g/L MS-222 (Sigma-Aldrich Corp., St. Louis, MO, USA), and individually weighed. Subsequently, a total of 375 selected juvenile largemouth bass with an initial body weight of 11.00 ± 0.20 g were randomly allocated into 15 experimental tanks (25 fish per tank) for an 8-week feeding trial. The tank has a height of 70 cm, with a circular base that has a radius of 30 cm. Each dietary treatment group was randomly assigned to 3 replicate tanks. Fish were fed to apparent satiation at 08:30 and 17:30 daily, with each feeding session lasting 15–20 min until satiation behavior was observed. Daily waste removal was performed using a siphon tube. During the trial, water temperature was maintained within the range of 28–30 °C, dissolved oxygen (DO) > 6 mg/L, and ammonia nitrogen < 0.2 mg/L. The determination of ammonia nitrogen and dissolved oxygen was conducted with the test kits provided by Sunpu Biochemical Co., Ltd. (Beijing, China), following the procedures specified in the accompanying instruction manual.

### 2.3. Sample Collection

At the end of the experiment, fish were fasted for 24 h and anesthetized with 0.1 g/L MS-222 (Sigma-Aldrich Corp., St. Louis, MO, USA) before sampling. Afterward, each fish was individually measured for body length and weight. Six fish per tank were randomly selected for blood collection via caudal venipuncture. Blood samples were stored at 4 °C. After allowing to stand overnight, samples were centrifuged at 3500 rpm for 15 min to harvest supernatants, which were then stored at −80 °C for intestinal permeability analysis. Subsequently, the fish were placed on an ice tray for dissection to isolate and weigh the liver, visceral mass, and intestinal fat, followed by measuring intestinal length and weight to facilitate subsequent calculation of morphometric indices. Then, two fish per tank were randomly selected for whole-body composition analysis. In addition, 4% paraformaldehyde was preprepared in 1.5 mL centrifuge tubes. Distal intestine from two fish per tank was collected and immersed in paraformaldehyde for intestinal histological analysis. Next, two fish were randomly selected from each tank to collect intestinal samples for the analysis of immune-related factors. The distal intestine of eight fish per tank was randomly selected for microbiome and transcriptome analysis. The distal intestines of each two fish were merged into one sample, of which two samples were used for microbiome analysis, and the other two samples were used for transcriptome analysis. Finally, two parallel distal intestine samples were collected for RT-qPCR analysis to validate the reliability of the transcriptome data. All collected samples, except tissue sections, were stored at −80 °C until further analysis. In addition, all the procedures were performed in compliance with the corresponding bioethical standards.

### 2.4. Chemical Analysis

The whole-body moisture, crude protein, and crude lipid of the experimental fish were determined following the AOAC standard method [38]. Briefly, samples were dried at 105 °C to constant weight for measuring whole-body moisture content; crude protein content was determined by the Kjeldahl method (N × 6.25) (Shanghai Qianjian Instruments Co., Ltd., Shanghai, China); crude lipid content was detected using the Soxhlet method (Shanghai Yuejin Medical Equipment Co., Ltd., Shanghai, China).

### 2.5. Intestinal Histological and Morphological Analysis

Intestinal tissue samples were sequentially processed as follows: dehydrated through a graded ethanol series, embedded in paraffin, sectioned at 4 μm thickness, and mounted on glass slides. Hematoxylin and eosin (H&E) staining was performed for histological evaluation. Intestinal pathological parameters were visualized under an optical microscope (ECLIPSE Ti-E, Nikon Corporation, Tokyo, Japan) and image acquisition software (NIS-Elements D 5.42, Nikon Corporation, Tokyo, Japan). The muscle layer thickness (MLT), plica height (PH) and plica width (PW) were measured by Image J software (Image J 1.53e, National Institutes of Health, Bethesda, MD, USA).

### 2.6. Intestinal Permeability Assessment

The assays for D-Lactic acid (D-LA, catalogue numbers: ml098823), diamine oxidase (DAO, catalogue numbers: ml076346), and lipopolysaccharide (LPS, catalogue numbers: ml093059) were performed using commercial kits (Shanghai Enzyme-linked Biotechnology Co., Ltd., Shanghai, China) according to the manufacturer’s instructions. Mix tissue and extraction solution at a ratio of 1 g tissue: 5–10 mL solution. Homogenize on ice, then centrifuge at 12,000× *g*, 4 °C; collect the supernatant and keep it on ice for subsequent testing. The general preparation process is similar across different kits, while specific details should be followed according to the instruction manual.

### 2.7. Physiological and Biochemical Analysis of the Intestine

The complement components in intestinal tissues (C3, catalogue numbers: ml003460; C4, catalogue numbers: ml003461) were analyzed using commercial kits from Shanghai Enzyme-linked Biotechnology Co., Ltd. (Shanghai, China). The Acid phosphatase (ACP, A060-2) and Alkaline phosphatase (AKP, A059-2) were assayed with commercial kits purchased from Nanjing Jiancheng Bioengineering Institute (Nanjing, China). Add the tissue to an appropriate amount of physiological saline and mash it. Centrifuge at 1000× *g* for 10 min, then collect the supernatant for subsequent testing.

### 2.8. Intestinal Microbial Analysis

Intestinal microbiota DNA was extracted with the HiPure Stool DNA Extraction Kit (Magen Biotechnology Co., Ltd., Guangzhou, China) according to the manufacturer’s instructions. The V3-V4 regions of the 16S rRNA gene were amplified via PCR using primers 341F (CCTACGGGNGGCWGCAG) and 806R (GGACTACHVGGGTATCTAAT). PCR products were then assessed for quality by 2% agarose gel electrophoresis, purified with AMPure XP Beads (Beckman Coulter, Inc., Indianapolis, IN, USA), and quantified using a Qubit 3.0. Libraries were constructed with the Illumina DNA Prep Kit (Illumina, Inc., San Diego, CA, USA) and validated on the ABI StepOnePlus Real-Time PCR System (Applied Biosystems, a brand of Life Technologies Corporation, Foster City, CA, USA). Validated libraries underwent sequencing on a Novaseq 6000 platform in PE250 mode.

Raw Illumina sequencing data were processed to obtain clean reads via filtering and merging using FASTP (v0.18.0) and FLASH (v1.2.11). Clean tags were clustered into OTUs (97% similarity) with Usearch (v11.0.667) through UPARSE, and chimeras were eliminated using UCHIME. Effective tags were used for OTU abundance analysis. Taxonomic annotations were performed against the SILVA database (v138.2), and abundance statistics at each taxonomic level were visualized with Krona (v2.6). Species abundance stacked plots were generated in R using the ggplot2 package. Diversity indices (Sob, Chao1, ACE, Shannon, Simpson) were calculated in R following formulas from the Mothur website (https://mothur.org/wiki/calculators/) Accessed on 15 November 2023. Principal coordinates analysis (PCoA) based on Bray–Curtis dissimilarity was conducted using the vegan package in R, with results visualized via ggplot2. Additionally, linear discriminant analysis (LDA) was used to identify taxa with significant abundance differences, based on their impact on sample composition.

### 2.9. Intestinal Transcriptomic Analysis

Total RNA was extracted from intestinal tissues with TRIzol reagent (Invitrogen, Carlsbad, CA, USA) following the manufacturer’s protocol. mRNA was then enriched using capture beads, purified, and fragmented by high-temperature treatment. The fragmented mRNA served as a template for first-strand cDNA synthesis via reverse transcription. During second-strand cDNA synthesis, end repair and A-tailing were completed. Adapters were subsequently ligated, and target fragments were selected and purified using Hieff NGS^®^ DNA Selection Beads. Finally, PCR amplification of the library was performed, followed by detection with the Illumina Novaseq X Plus.

High-quality quality clean reads were obtained by filtering with FASTP (v0.18.0). Reads were then mapped to the ribosome RNA (rRNA) database using Bowtie2 (v2.2.8), and rRNA mapped reads were removed. The remaining clean reads were used for assembly and gene abundance calculation. Principal component analysis (PCA) was conducted with the R package (Version 4.2.1) gmodels (http://www.r-project.org/) Accessed on 4 November 2023. Differential RNA expression between groups was analyzed using DESeq2, with genes considered differentially expressed if they met the criteria of false discovery rate (FDR) < 0.05 and absolute fold change ≥ 2. Subsequent GO and KEGG enrichment analyses were performed on these differentially expressed genes (DEGs). For GO analysis, all DEGs were mapped to terms in the Gene Ontology database (http://www.geneontology.org/) Accessed on 4 November 2023., gene counts per term were calculated, and significantly enriched terms (relative to the genome background) were identified via hypergeometric testing. For KEGG analysis, significantly enriched metabolic or signal transduction pathways in DEGs (vs. the whole genome background) were determined. *p*-values were corrected for FDR, with FDR ≤ 0.05 as the threshold for defining significant enrichment.

### 2.10. Quantitative PCR

Total RNA was extracted from intestinal samples using the TRIzol method with a commercial kit. Subsequently, cDNA synthesis was performed using a reverse transcription kit (RR092A; Takara Bio Inc., Kusatsu, Japan). Then, quantitative real-time PCR was performed using CFX96 Touch™ Real-Time PCR detection system (Bio-Rad, Hercules, CA, USA) with the thermal cycling program set as follows based on the characteristics of the designed primers and PCR enzymes: 95 °C for 30 s (pre-denaturation), followed by 40 cycles of 95 °C for 5 s (denaturation) and 57 °C for 30 s (annealing). Each qPCR reaction was conducted in technical replicates using gene-specific primers for *plb1*, *cd36*, *acsl5*, *jun*, *il22*, and *eef1a1*, with primer sequences detailed in Table 2. *eef1a1* was used as the reference gene [39]. Finally, the gene expression level was calculated using the 2^−ΔΔCt^ method [40].

### 2.11. Calculations

The formulas for calculating growth performance parameters, feed utilization efficiency, and morphometric indices are as follows, where IBW denotes initial body weight and FBW denotes final body weight.

$$\begin{array}{c} WGR\;(Weight\;gain\;rate,\%)=[(FBW-IBW)/IBW]\times 100\\ SGR\;(Spectific\;growth\;rate,\%/d)=[ln(FBW)-ln(IBW)]/[experimental\;period(d)]\times 100\\ PER\;(Protein\;efficiency\;ratio)=[FBW(g)-IBW(g)]/[dry\;feed\;intake(g)\times dietary\;protein\;content]\\ FCR\;\left(Feed\;conversion\;ratio\right)=dry\;feed\;consumed\;(g)/[FBW\;(g)-IBW\;(g)]\\ FI\;(Feed\;intake,\%/d)=[dry\;feed\;intake\;(g)/experimental\;period\;(d)]/[(FBW-IBW)/2]\times 100\\ SR\;(Survival\;rate,\%)=number\;of\;fish\;survival/initial\;fish\;number\times 100\\ VSI\;\left(Visceral\;somatic\;index,\%\right)=Visceral\;weight\;(g)/body\;weight\;(g)\times 100\\ HSI\;\left(Hepatosomatic\;index,\%\right)=hepatopancreas\;weight\;(g)/body\;weight\;(g)\times 100\\ AFR\;\left(Abdominal\;fat\;rate,\%\right)=abdominal\;fat\;weight\;(g)/body\;weight\;(g)\times 100\\ ISI\;\left(Intestinal\;weight\;index,\%\right)=intestinal\;weight\;(g)/body\;weight\;(g)\times 100\\ ILI\;\left(Intestinal\;length\;index,\%\right)=intestinal\;length\;(cm)/body\;length\;(cm)\times 100 \end{array}$$

### 2.12. Statistical Analysis

Before conducting one-way analysis of variance (One-way ANOVA), the normality of data distribution was verified via the Shapiro–Wilk test, and the homogeneity of variances was assessed using Levene’s test. Then, One-way ANOVA was performed using SPSS statistical software (Version 27; IBM, Armonk, NY, USA), followed by Duncan’s multiple range test for post hoc comparisons among groups. *p* < 0.05 was considered a significant difference. Subsequently, figures were plotted using GraphPad Prism 8 (San Diego, CA, USA). All data are presented as mean ± standard error of the mean (SEM). To determine whether the effect showed a linear and/or quadratic relationship, trend analysis was performed using orthogonal polynomial contrast in SPSS 27.

## 3. Results

### 3.1. Growth Performance

There were no significant differences in the FBW, WGR, SGR, PER, FCR, and FI among the groups (*p* > 0.05; Table 3). With the level of rapeseed meal replacing fish meal increased to 25%, the growth performance indices showed a decreasing trend, except for FCR and FI (*p* > 0.05). The survival rate of each group was 100%.

### 3.2. Morphology Indices

No significant differences were detected in the VSI, AFR, ISI, and ILI in any of the groups (*p* > 0.05; Table 4). However, the FM and RM5 groups had significantly higher HSI compared with the other groups (*p* < 0.05). The relationship between RM replacing levels and the dependent variable of HSI was better described by a linear model or a quadratic model.

### 3.3. Whole-Body Composition

There were no significant differences in moisture content in any of the groups (*p* > 0.05; Table 5). However, the crude lipid and crude protein contents of body composition were significantly influenced by RM replacing levels (*p* < 0.05). Groups FM and RM5 had significantly higher crude lipid and crude protein contents compared with the other groups (*p* < 0.05). The relationship between RM replacing levels and both crude lipid and crude protein was better described by a linear model or a quadratic model.

### 3.4. The Intestinal Structure

**Figure 1:**
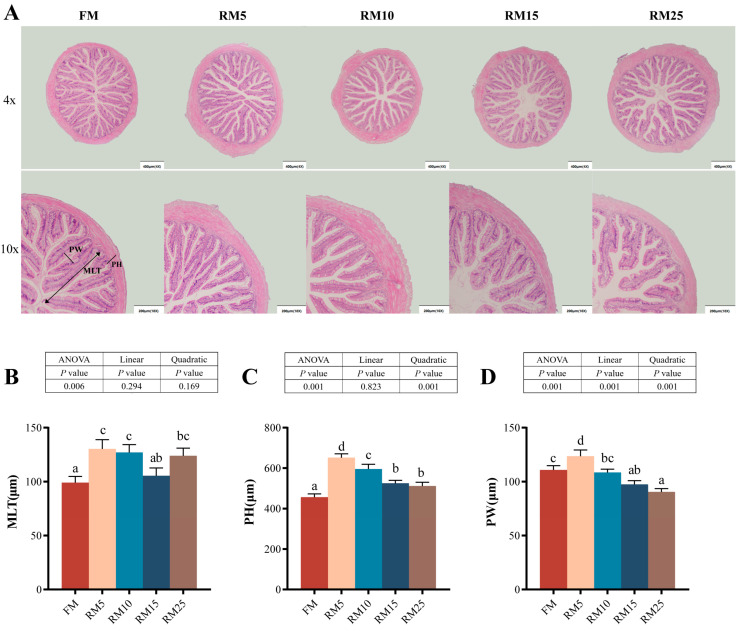
Effects of replacing fish meal with rapeseed meal on the intestinal histological structure of largemouth bass. (**A**) Intestinal histological structure by H&E (scale bars: 400 and 200 μm). (**B**) MLT: muscle layer thickness. (**C**) PH: plica height. (**D**) PW: plica width. The data are all expressed as mean ± SEM (*n* = 3). Significant differences among all the data were represented by different letters in the columns (*p* < 0.05). A linear or quadratic trend analysis described the response of replacing fish meal with rapeseed meal using orthogonal polynomial contrasts.

The muscle layer thickness (MLT), plica height (PH), and plica width (PW) were significantly influenced by RM replacing levels (*p* < 0.05; Figure 1). Groups RM5 and RM10 had significantly higher MLT compared with the FM group (*p* < 0.05). In addition, the RM5 groups showed significantly higher PH and PW values compared with the other groups (*p* < 0.05). The relationship between RM replacing levels and both PH and PW was better described by a quadratic model.

### 3.5. Liver Histology Structure

**Figure 2:**
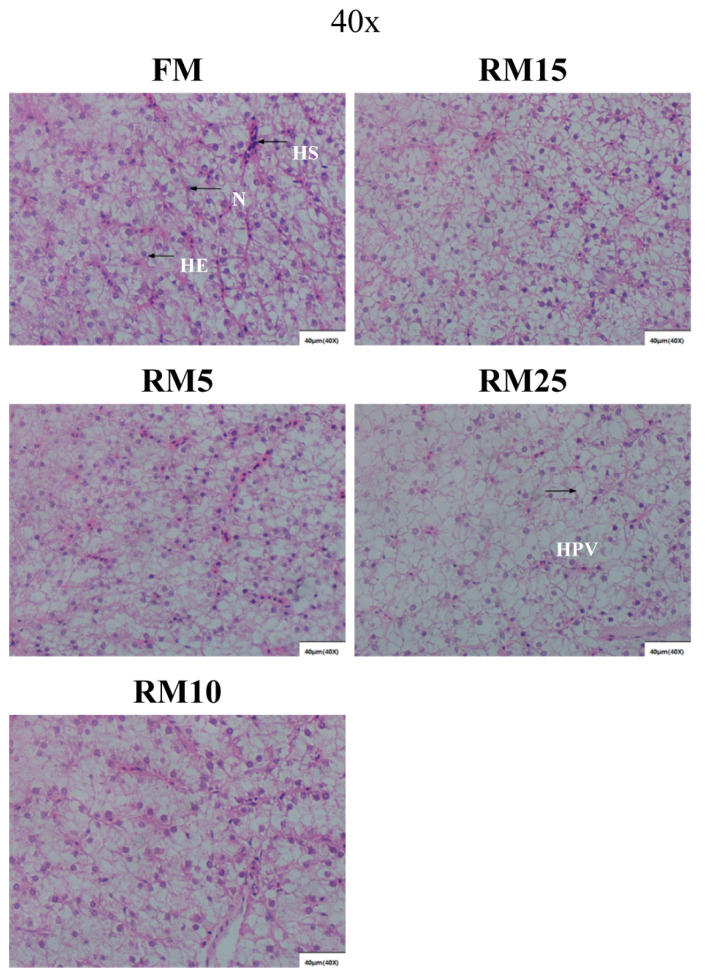
Effects of replacing fish meal with rapeseed meal on the liver of largemouth bass (scale bars: 40 μm). He: hepatocyte; N: nucleus; HS: hepatic sinusoid; HPV: hepatocyte vacuolation.

The results of liver tissue sections showed that with the increase of rapeseed meal replacement level, the number of hepatocytes, nuclei, and nucleus sinusoids gradually decreased, and vacuolization appeared in the tissues, especially in the RM25 group (Figure 2). Liver damage may interfere with the metabolic capacity of largemouth bass, thereby inducing a series of negative effects caused by nutrient absorption issues.

### 3.6. The Intestinal Permeability

**Figure 3:**
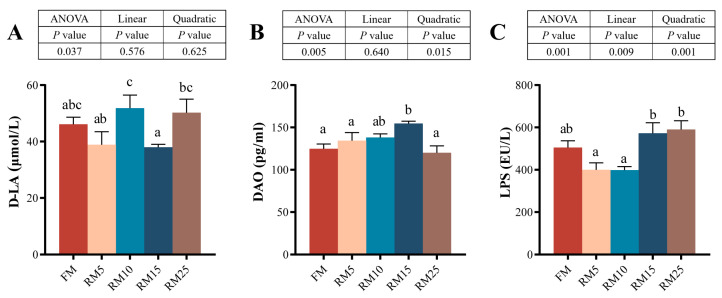
Effects of replacing fish meal with rapeseed meal on D-LA, DAO, and LPS in largemouth bass. (**A**) D-LA: D-Lactic acid. (**B**) DAO: diamine oxidase. (**C**) LPS: lipopolysaccharide. The data are all expressed as mean ± SEM (*n* = 3). Significant differences among all the data were represented by different letters in the columns (*p* < 0.05). A linear or quadratic trend analysis described the response of replacing fish meal with rapeseed meal using orthogonal polynomial contrasts.

The results showed that the contents of D-lactic acid (D-LA), diamine oxidase (DAO), and lipopolysaccharide (LPS) were significantly influenced by the RM replacing levels (*p* < 0.05; Figure 3). D-LA and LPS levels in the RM25 group were higher than those in the FM group. Additionally, the DAO level in the RM15 group also showed a significantly high value (*p* < 0.05). The relationship between RM replacing levels and LPS was better described by a linear model or a quadratic model. In contrast, the relationship between DAO and RM replacing levels only shows a significant quadratic model.

### 3.7. The Intestinal Physiological and Biochemical Analysis

**Figure 4:**
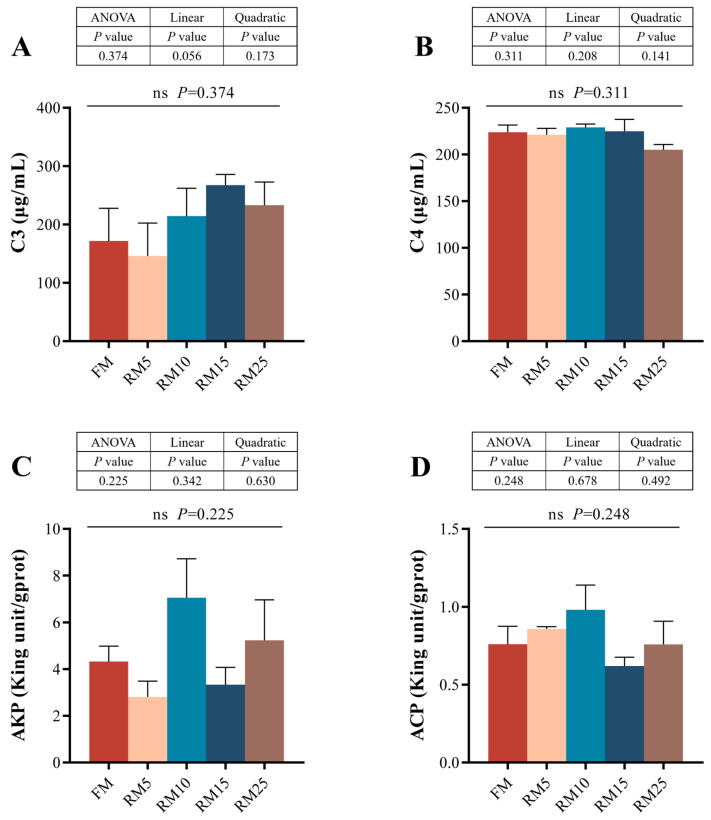
Effects of replacing fish meal with rapeseed meal on intestinal immune factors in largemouth bass. (**A**) C3: complement component 3. (**B**) C4: complement component 4. (**C**) ACP: acid phosphatase. (**D**) AKP: alkaline phosphatase. The data are all expressed as mean ± SEM (*n* = 3). and ns means that there is no significant difference. A linear or quadratic trend analysis described the response of replacing fish meal with rapeseed meal using orthogonal polynomial contrasts.

No significant differences in C3, C4, AKP, or ACP were observed among any of the groups (*p* > 0.05, Figure 4). Moreover, these indices did not exhibit linear or quadratic trends with the decrease in fish meal proportion in the diet (*p* > 0.05).

### 3.8. Analysis of the Intestinal Microbiota

**Figure 5:**
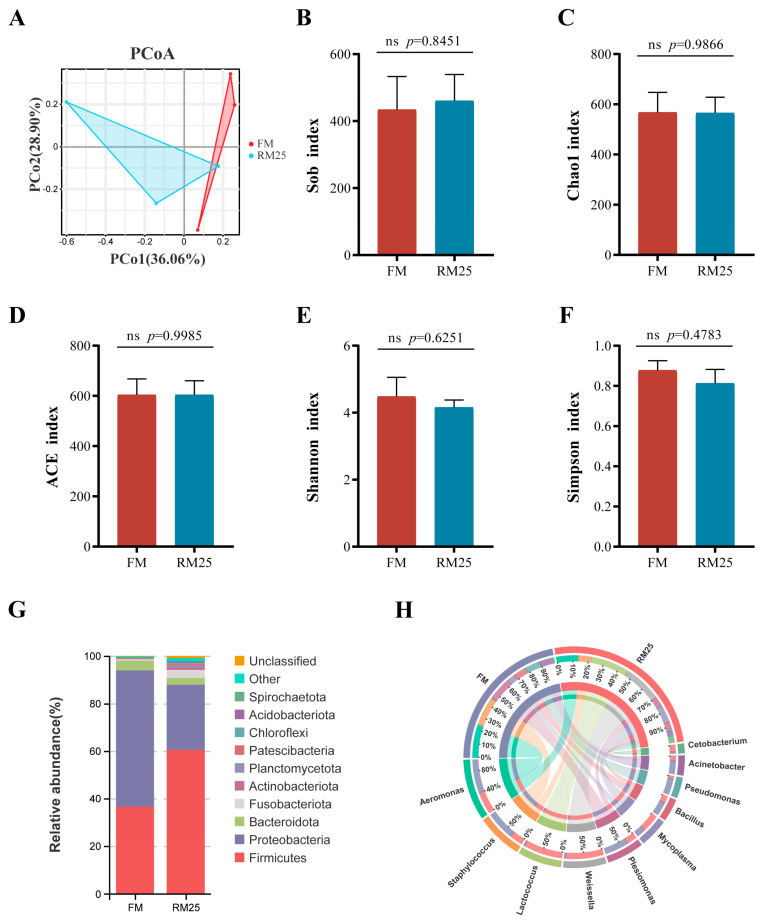
Effects of replacing fish meal with rapeseed meal on intestinal microbiota of largemouth bass. (**A**) Principal coordinate analysis (PCoA) based on Bray–Curtis distance at the OTU level. (**B**) Sob index. (**C**) Chao1 index. (**D**) ACE index. (**E**) Shannon index. (**F**) Simpson index. (**G**) major species composition at the phylum level. (**H**) major species composition at the genus level. The data are all expressed as mean ± SEM (*n* = 3). as determined by an independent *t*-test, and ns means that there is no significant difference between the two groups.

As the results showed in Figure 5, the differences and distances between the FM and RM25 groups were reflected by PcoA. The contribution rates of Pco1 and Pco2 were 36.6% and 28.90%, respectively (Figure 5A). Although there were no significant differences in the alpha diversity indices (Sob, Chao1, ACE, Shannon, and Simpson) between groups (*p* > 0.05). The Shannon index and Simpson index in the RM25 group were lower than those in the FM group, although there is no statistical difference (Figure 5B–F).

At the phylum level, Firmicutes, Proteobacteria, Bacteroidota and Fusobacteriota were the top four dominant microbiota in the FM and RM25 groups. The relative abundances of Proteobacteria and Bacteroidota in the FM group were higher than those in the RM25 group. However, the relative abundances of Firmicutes and Fusobacteriota in the RM25 group were higher than those in the FM group (Figure 5G). At the genus level, *Aeromonas*, *Staphylococcus*, *Plesiomonas*, *Pseudomonas* and *Bacillus* were the top five genera with relatively high abundances in the FM group. But in the RM25 group, *Lactococcus*, *Weissella*, *Aeromonas*, *Mycoplasma* and *Staphylococcus* were the top five genera (Figure 5H).

**Figure 6:**
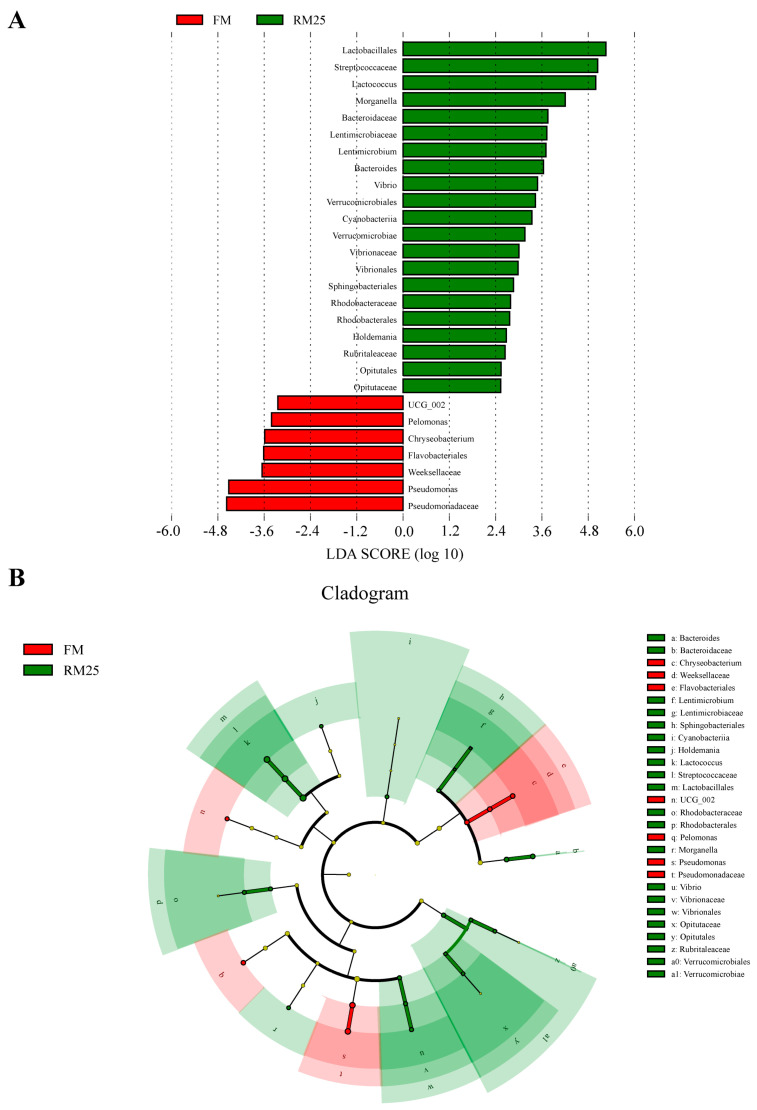
LEfSe analysis of the differences in the intestinal microbiota of largemouth bass between the two groups. (**A**) LEfse distribution histogram with a threshold of LDA > 2. (**B**) Clogram based on LEfse analysis.

LEfse analysis revealed significant differences (LDA > 2, *p* < 0.05) in the intestinal microbiota between FM and RM25 (Figure 6). The results showed that the relative abundances of Lactobacillales, Streptococcaceae, *Lactococcus* and *Morganella* in the RM25 group were significantly increased compared with those in the FM group. However, the relative abundances of Pseudomonadaceae, Weeksellaceae, Flavobacteriales and *Pseudomonas* were significantly decreased in the RM25 group compared with those in the FM group.

### 3.9. Analysis of the Intestinal Transcriptome

**Figure 7:**
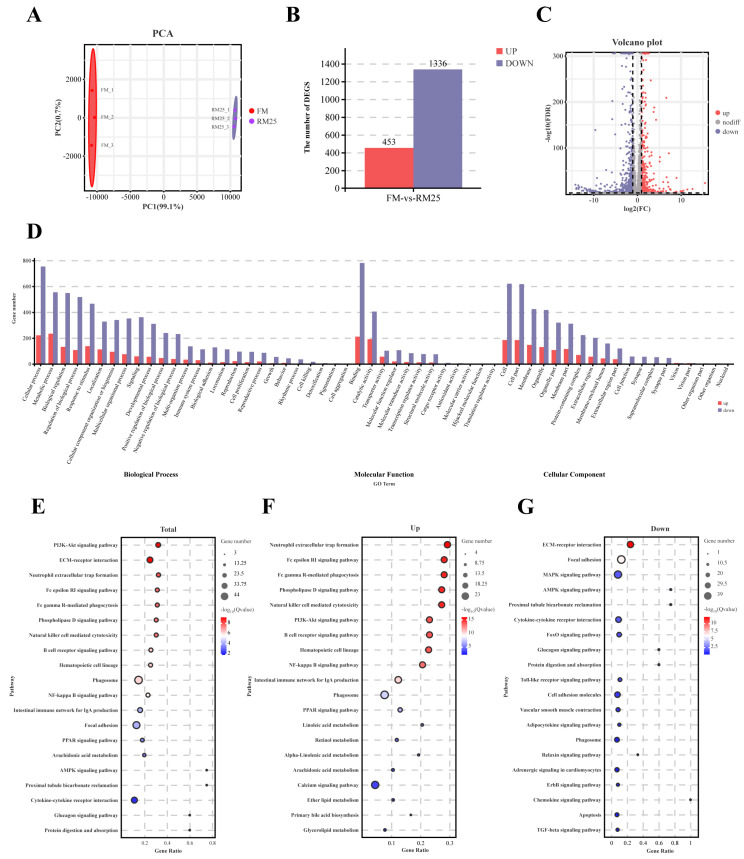
Analysis of the intestinal transcriptome of largemouth bass in the FM and RM25 groups. (**A**) Principal component analysis between the FM and RM25 groups. (**B**) Column chart of the number of DEGs. Up-regulated differentially expressed genes are represented by red color, and down-regulated differentially expressed genes are represented by blue color. (**C**) Volcano plot of the distribution of DEGS. (**D**) The enriched Gene Ontology (GO) terms of the DEGs. The *X*-axis corresponds to the GO terms; the *Y*-axis indicates the number of DEGs for each GO term. (**E**) The top 20 enriched Kyoto Encyclopedia of Genes and Genome (KEGG) pathways of all DEGs. (**F**) The top 20 enriched Kyoto Encyclopedia of Genes and Genome (KEGG) pathways of upregulated DEGs. (**G**) The top 20 enriched Kyoto Encyclopedia of Genes and Genome (KEGG) pathways of down-regulated DEGs.

PCA analysis showed that there were obvious differences between the two groups (Figure 7A). It can be seen that there was a total of 1789 DEGs between the two groups from the column chart. Among them, 453 DEGs were upregulated and 1336 were downregulated (Figure 7B). The volcano plot displays the distribution of each DEG (Figure 7C). Then, Gene Ontology (GO) enrichment analysis was conducted on all DEGs and is shown in Figure 7D. The GO terms are divided into three parts: biological process, molecular function, and cellular component. In the biological process category, the DEGs are mainly enriched in cellular process, metabolic process, biological regulation, and response to stimulus; In the molecular function category, they are mainly enriched in binding and catalytic activity; In the cellular component category, they are mainly enriched in cell, cell part, membrane, organelle, etc. Finally, Kyoto Encyclopedia of Genes and Genome (KEGG) enrichment analysis showed the top 20 pathways enriched with DEGs, which are presented in Figure 7E–G. The results showed that the Neutrophil extracellular trap formation, PI3K-Akt, and NF-κB signaling pathways were significantly upregulated in the RM25 and FM comparison groups.

### 3.10. Verification of the Reliability of Transcriptome Data by qPCR

**Figure 8:**
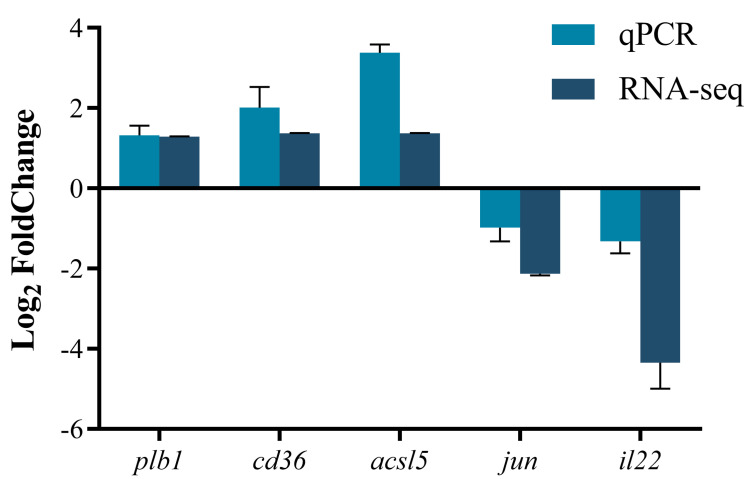
Verification of the reliability of the transcriptome data by qPCR. The qPCR analysis was performed on several candidate genes related to immune stress in the intestinal tissues of largemouth bass. The relative expression levels of the genes are presented with log_2_(FoldChange) on the y axis. *Plb1*: phospholipase B1; *cd36*: CD36 molecule (thrombospondin receptor); *acsl5*: acyl-CoA synthetase long chain family member 5; *jun*: Jun proto-oncogene; *il22*: interleukin 22.

In order to verify the reliability of the transcriptome data, the qPCR technique was used to validate the expression levels of several randomly selected genes. The results are shown in Figure 8. The upregulation levels of the *cd36* and *acsl5* genes in qPCR were higher than those in RNA-seq, while the downregulation levels of the *jun* and *il22* genes in qPCR were lower than those in RNA-seq. The expression of *Plb1* was comparable between the two methods, and the overall change trend was consistent with the results of RNA-seq.

## 4. Discussion

### 4.1. Effects of Diets Containing Rapeseed Meal on the Growth Performance of Largemouth Bass

Rapeseed protein is processed to produce rapeseed meal, which has exhibited good performance in the aquaculture of various fish species. A rearing experiment on red sea bream showed that when rapeseed meal replaced fish meal to provide 50% of the protein, the FBW, WGR, SGR, and PER of red sea bream were not significantly affected [41]. Similarly, this study found that replacing up to 25% of fish meal with rapeseed meal had no significant negative effects on the FBW, WGR, SGR, and PER of largemouth bass. However, the downward trend of these indicators in the RM groups may be attributed to several factors, including antinutritional factors (ANFs), lower digestibility, and lower nutrient contents. These factors can impair nutrient utilization and reduce the growth performance of largemouth bass. For carnivorous fish, feeding plant protein may lead to reduced feed intake due to decreased palatability and undesirable taste [42]. Nevertheless, the FI of the RM groups was not negatively affected. Similar results supporting this finding have also been observed in other fish species. In the diet of juvenile *Pseudobagrus ussuriensis* [43], an increase in the substitution level of soybean meal did not affect its FI. Additionally, Kokou et al. [44] reported that feed consumption is not affected by the substitution level. This may be attributed to the low nutrient absorption rate of diets with high-level rapeseed meal replacing fish meal, leading largemouth bass to increase in feed intake to meet the nutritional requirements for growth.

### 4.2. Effects of Diets Containing Rapeseed Meal on the Morphological Indicators and Whole-Body Composition of Largemouth Bass

Morphological parameters such as HSI and VSI can effectively reflect the nutritional status of fish and serve as indicators of physiological conditions [45]. In this study, the HSI of the FM and RM5 groups was significantly higher than that of the other groups. The results indicated that the nutritional status of fish gradually deteriorated as the level of rapeseed meal replacing fish meal increased to 25%. However, it is contrary to the results observed in tilapia (*Oreochromis niloticus*) [46] and cobia (*Rachycentron canadum*) [47]. The underlying mechanisms of this discrepancy remain unclear, but the decrease in HSI may be due to an amino acid imbalance in dietary proteins, which could lead to the decomposition of certain amino acids for lipid synthesis [48]. Additionally, Groups FM and RM5 had significantly higher crude lipid and crude protein contents compared with the other groups. This observation aligns with the findings reported by Dossou et al. [49] and Li et al. [46].

### 4.3. Effects of Diets Containing Rapeseed Meal on the Liver Structure of Largemouth Bass

The liver is a vital metabolic organ in fish. In our study, feeding largemouth bass with rapeseed meal-containing diets was found to cause a reduction in hepatocyte count to some extent, and even severe vacuolization was observed in the RM25 group. Previous studies have shown that excessive intake of plant protein increases the metabolic burden on fish livers and even causes liver damage [50]. Rapeseed meal contains glucosinolates that can decompose into metabolites such as thiocyanate, isothiocyanate, and oxazolidinethione under certain conditions. These hydrolyzed glycosides are considered toxic and difficult to degrade in fish, potentially causing hepatocyte enlargement and fragmentation [51] and leading to vacuolization in the liver. Therefore, feeding largemouth bass with rapeseed meal-containing diets may lead to liver damage.

### 4.4. Effects of Diets Containing Rapeseed Meal on the Intestinal Structure and Biochemical Indicators of Largemouth Bass

The intestine of fish is a vital organ in direct contact with food. It not only functions in digesting and absorbing nutrients but also serves as a barrier to prevent harmful substances, such as bacteria, from entering organs and tissues [52]. The intestinal plicae are the primary sites for the digestion and absorption of nutrients [53]. It has been reported in other fish species that feeding on plant proteins impairs the intestinal structure of fish and reduces MLT and PH [54,55,56]. Interestingly, Groups RM5 and RM10 had significantly higher MLT compared with the FM group. Moreover, the RM5 groups showed significantly higher PH and PW values compared with the other groups. We speculated that feeding diets containing rapeseed meal may stimulate the intestine to better absorb nutrients. The specific mechanisms require further investigation in future studies. In addition, Previous studies have demonstrated that the complement system plays an indispensable role in maintaining intestinal barrier function and initiating immune responses [57]. Complement factors can be directly detected in the intestinal lumen and are capable of regulating the functions of intestinal epithelial cells [58]. In our study, the intestinal immunity-related indices C3, C4, AKP, and ACP, no significant differences, which is consistent with the above-mentioned growth performance results of largemouth bass. The function of the intestine depends on the integrity of the intestinal structure [59]. When damage occurs to intestinal epithelial cells or the tight junction layer, the serum levels of both D-LA and DAO increase [60]. Therefore, monitoring serum levels of D-LA and DAO can be used to reflect changes in intestinal mucosal integrity and permeability [61]. In this study, D-LA and LPS in the RM25 group were higher than in the FM group. Additionally, the DAO level in the RM15 group also showed a significantly high value. Thus, our results support the view that replacing fish meal with rapeseed meal in the diet disrupts the intestinal structural integrity in largemouth bass.

### 4.5. Effects of Diets Containing Rapeseed Meal on the Intestinal Microbiota of Largemouth Bass

Fish intestinal microbiota is a key factor in regulating nutrient digestion, immune responses, intestinal differentiation, and disease resistance [62]. Furthermore, the intestinal microbiota is closely associated with fish species, growth stage, dietary composition, and feeding environment [63]. Dietary components influence biological changes in fish by altering their intestinal microbiota [64]. In this study, the results showed no significant differences in the Sob, Chao1, ACE, Shannon, and Simpson indices, indicating that there were no obvious differences in the diversity and abundance of intestinal microbiota between the two groups. Similar results have been observed in other studies [65,66]. Lin et al. [67] reported that the intestinal microbiota of largemouth bass is dominated by Firmicutes, Proteobacteria, and Fusobacteriota. Likewise, we also observed similar results. This study showed that the RM25 group had a higher relative abundance of Firmicutes, while the FM group showed a higher relative abundance of Proteobacteria. Firmicutes are considered a beneficial phylum as they can produce butyrate-based metabolites. As the primary energy source for intestinal epithelial cells, butyrate plays a crucial role in preventing intestinal inflammation [68]. Additionally, Firmicutes can promote cellulose decomposition and polysaccharide fermentation in the intestine [69]. Therefore, we infer that when feeding diets containing plant proteins, the intestine generates more relevant microbial communities to digest cellulose. A study on turbot (*Scophthalmus maximus*) has shown that replacing fish meal with soybean meal increases the relative abundance of Firmicutes [70]. Proteobacteria are regarded as indicators of microbiota dysregulation and the emergence of diseases [71]. They primarily use protein as an energy source, which may lead to host metabolic disorders [72]. At the genus level, the RM25 group exhibited higher levels of *Lactococcus* and *Weissella* and lower levels of *Aeromonas* and *Staphylococcus* compared to the FM group. *Lactococcus* and *Weissella* are associated with disease resistance and improved immunity in fish [73,74], while *Aeromonas* and *Staphylococcus* may cause intestinal inflammation and even death in fish [75,76]. In summary, Rapeseed meal substitution for fish meal altered the composition of the intestinal microbiota in largemouth bass.

### 4.6. Effects of Diets Containing Rapeseed Meal on the Gene Expression of Largemouth Bass

The KEGG enrichment analysis showed that upregulated DEGs were predominantly enriched in Neutrophil extracellular trap formation, PI3K-Akt signaling, and NF-κB signaling pathways. As is well known, Neutrophils are universally present in most mammals and play critical roles in innate immune defense, inflammatory responses, and tissue injury [77]. As classical inflammatory cells, neutrophils can efficiently clear pathogens and cellular debris from postcapillary venules [78]. However, neutrophils may excessively accumulate at inflamed sites, exacerbating tissue damage [79]. Additionally, the NF-κB signaling pathway is involved in the development of intestinal inflammation. Overall, the results may be associated with the impaired intestinal structure in response to rapeseed meal replacing fish meal.

## 5. Conclusions

The replacement of 5% fish meal with rapeseed meal did not have a negative impact on the physiological status of largemouth bass. However, a replacement level of 25% reduced growth performance and damaged intestinal structure, potentially by altering the abundance of intestinal microbiota and up-regulating PI3K-Akt and NF-κB signaling pathways. This study provides new insights into the application of rapeseed meal in carnivorous fish.

## Figures and Tables

**Table 1:** Formulation and proximate composition of the experimental diets (% dry matter).

Ingredients (%)	FM	RM5	RM10	RM15	RM25
	0	5%	10%	15%	25%
Fish meal	42.00	39.90	37.80	35.70	31.50
Rapeseed meal ^1^	0.00	3.51	7.01	10.52	17.53
Chicken meal	13.00	13.00	13.00	13.00	13.00
Plasma protein meal	5.00	5.00	5.00	5.00	5.00
Pork meal	5.00	5.00	5.00	5.00	5.00
Peanut meal	6.00	6.00	6.00	6.00	6.00
Wheat flour	12.00	12.00	12.00	12.00	11.82
Fish oil	2.00	2.10	2.30	2.50	2.70
Soybean oil	2.00	2.00	2.00	2.00	2.00
Calcium dihydrogen phosphate	1.50	1.50	1.50	1.50	1.50
Choline chloride	0.50	0.50	0.50	0.50	0.50
Vitamin C	0.10	0.10	0.10	0.10	0.10
Vitamin and mineral premix	2.00	2.00	2.00	2.00	2.00
Ethoxyquin	0.05	0.05	0.05	0.05	0.05
Lysine ^2^	0.02	0.65	0.75	0.85	1.05
Methionine ^2^	0.17	0.19	0.28	0.22	0.25
Bentonite	4.33	3.25	2.36	1.53	0.00
Microcrystalline cellulose	4.33	3.25	2.36	1.53	0.00
Total	100	100	100	100	100
Proximate composition (%)					
Crude protein	48.51	49.16	49.35	49.39	49.59
Crude lipid	10.28	10.22	10.25	10.28	10.14
Crude ash	8.19	8.23	8.34	8.42	8.36
Moisture	8.88	8.24	7.40	7.95	7.74

^1^ Rapeseed meal: crude protein 37.5%, crude lipid 1.53%, crude ash 7.9%. ^2^ Lysine and Methionine were purchased from Shanghai Sanjie Biotech Co., Ltd., Shanghai, China. All other ingredients were purchased from Chongqing CITICO Biotech Co., Ltd., Chongqing, China.

**Table 2:** Primer sequences for qPCR.

Gene	Primer Sequence (5′-3′)	Product Size (bp)	GenBank
*eef1a1*	F: GAAGCTCGAAGACAACCCCA R: TCACGGACTGCAAATCTCCC	129	XM_038714535.1
*il22*	F: GGGCGAGCGAGGTATAAACA R: GTGGCGGTGGAGTTTTTCAG	96	XM_038709272.1
*plb1*	F: GCTCAGCTTACAGACACGGT R: GTGAACTGAAGAGGACGGGG	132	XM_038729844.1
*cd36*	F: TGCTGTAACAGAAGGTGCGG R: CAGGCTCAATGATGACTTCCTTC	136	XM_038739146.1
*jun*	F: GCAC AGAGAGGACGTTTGGA R: GCCGGCGTTGTCGTGTTTTA	114	XM_038716392.1
*acsl5*	F: TACCCTTACTGTGTGTGCTCC R: AGATAAACATCCTTCACCTGCTCA	141	XM_038736141.1

*eef1a1*: eukaryotic translation elongation factor 1-alpha 1; *il22*: interleukin 22; *plb1*: phospholipase B1; *cd36*: CD36 molecule (thrombospondin receptor); *jun*: jun proto-oncogene; *acsl5*: acyl-CoA synthetase long chain family member 5.

**Table 3:** Effects of replacing fish meal with rapeseed meal on the growth performance of Largemouth bass.

Items	FM	RM5	RM10	RM15	RM25	ANOVA	Linear	Quadratic
FBW (g)	47.45 ± 1.52	45.87 ± 0.77	45.45 ± 2.74	46.90 ± 2.11	43.40 ± 1.83	0.626	0.233	0.485
WGR (%/d)	331.17 ± 13.54	317.02 ± 7.40	313.73 ± 24.59	326.12 ± 18.89	294.54 ± 15.84	0.617	0.224	0.469
SGR (%)	2.61 ± 0.06	2.55 ± 0.03	2.53 ± 0.11	2.59 ± 0.08	2.45 ± 0.07	0.598	0.209	0.447
PER	1.81 ± 0.04	1.76 ± 0.04	1.82 ± 0.06	1.77 ± 0.06	1.77 ± 0.07	0.892	0.681	0.922
FCR	1.15 ± 0.03	1.18 ± 0.03	1.19 ± 0.04	1.19 ± 0.04	1.26 ± 0.05	0.453	0.072	0.186
FI (%/d)	2.57 ± 0.04	2.57 ± 0.04	2.59 ± 0.06	2.62 ± 0.04	2.66 ± 0.05	0.637	0.102	0.248
SR (%)	100 ± 0.00	100 ± 0.00	100 ± 0.00	100 ± 0.00	100 ± 0.00	-	-	-

IBW: initial body weight; FBW: final body weight; WGR: weight gain rate; SGR: specific growth rate; PER: protein efficiency ratio; FCR: feed conversion ratio; FI: feed intake; SR: survival rate. The data are all expressed as mean ± SEM (*n* = 3). A linear or quadratic trend analysis described the response of replacing fish meal with rapeseed meal using orthogonal polynomial contrasts.

**Table 4:** Effects of replacing fish meal with rapeseed meal on morphology indexes of Largemouth bass.

Items	FM	RM5	RM10	RM15	RM25	ANOVA	Linear	Quadratic
VSI (%)	8.21 ± 0.33	7.85 ± 0.23	7.61 ± 0.31	7.37 ± 0.25	7.78 ± 0.25	0.290	0.124	0.103
HSI (%)	2.46 ± 0.11 ^b^	2.69 ± 0.10 ^b^	2.10 ± 0.11 ^a^	1.96 ± 0.07 ^a^	2.08 ± 0.12 ^a^	0.001	0.001	0.001
AFR (%)	1.85 ± 0.13	1.64 ± 0.12	1.66 ± 0.12	1.63 ± 0.10	1.74 ± 0.11	0.682	0.540	0.362
ISI (%)	0.62 ± 0.03	0.61 ± 0.02	0.64 ± 0.03	0.71 ± 0.06	0.70 ± 0.02	0.113	0.014	0.049
ILI (%)	78.24 ± 2.06	79.38 ± 1.15	79.01 ± 1.49	74.73 ± 2.20	76.38 ± 1.74	0.308	0.137	0.303

VSI: visceral Somatic index; HSI: hepatosomatic index; AFR: abdominal fat rate; ISI: intestinal somatic index; ILI: intestinal length index. Means with different superscripts in the same line are significantly different (*p* < 0.05). The data are all expressed as mean ± SEM (*n* = 3). A linear or quadratic trend analysis described the response of replacing fish meal with rapeseed meal using orthogonal polynomial contrasts.

**Table 5:** Effects of replacing fish meal with rapeseed meal on the whole-body composition of Largemouth bass (% dry matter).

Items (%)	FM	RM5	RM10	RM15	RM25	ANOVA	Linear	Quadratic
Moisture	72.78 ± 11.29	71.75 ± 8.79	80.13 ± 0.45	79.40 ± 0.74	77.75 ± 2.01	0.832	0.338	0.582
Crude Lipid	8.13 ± 0.61 ^b^	7.80 ± 0.33 ^b^	5.92 ± 0.02 ^a^	5.58 ± 0.11 ^a^	6.19 ± 0.29 ^a^	0.010	0.006	0.006
Crude Protein	15.79 ± 0.16 ^c^	17.36 ± 0.09 ^d^	11.74 ± 0.00 ^a^	12.71 ± 0.07 ^b^	12.81 ± 0.18 ^b^	0.001	0.003	0.010

Means with different superscripts in the same line are significantly different (*p* < 0.05). The data are all expressed as mean ± SEM (*n* = 3). A linear or quadratic trend analysis described the response of replacing fish meal with rapeseed meal using orthogonal polynomial contrasts.

## Data Availability

The data that support the findings of this study are available from the corresponding author upon reasonable request.

## Abbreviation

**Full Name**	**Abbreviation**
Fish meal	FM
Rapeseed meal	RM
Muscle layer thickness	MLT
Plica height	PH
Plica width	PW
D-Lactic acid	D-LA
Diamine oxidase	DAO
Lipopolysaccharide	LPS
Acid phosphatase	ACP
Alkaline phosphatase	AKP
Initial body weight	IBW
Final body weight	FBW
Weight gain rate	WGR
Specific growth rate	SGR
Protein efficiency ratio	PER
Feed conversion ratio	FCR
Feed intake	FI
Survival rate	SR
Visceral somatic index	VSI
Hepatosomatic index	HSI
Abdominal fat rate	AFR
Intestinal weight index	ISI
Intestinal length index	ILI
Antinutritional factors	ANFs
